# Simulation and Experimental Study of the Isothermal Thermogravimetric Analysis and the Apparent Alterations of the Thermal Stability of Composite Polymers

**DOI:** 10.3390/polym16111454

**Published:** 2024-05-21

**Authors:** Costas Tsioptsias, Alexandros K. Zacharis

**Affiliations:** Department of Chemical Engineering, University of Western Macedonia, 50132 Kozani, Greece; azx15528@gmail.com

**Keywords:** thermal stability, polymer, composite, thermogravimetric analysis

## Abstract

Composite polymers are an interesting class of materials with a wide range of applications. Among the properties of polymers which are currently being enhanced via the development of composite materials is their thermal stability, which is typically evaluated via thermogravimetric analysis (TGA). In this work, a paradox is recognized regarding the considered relationship between the polymer–filler interactions leading to a good dispersion of the filler and the improvement of thermal stability. Simulation of the TGA signal during isothermal measurements of composite polymers is performed along with experimental measurements. It is shown that there are at least three factors that can cause apparent alterations of the thermal stability of composite polymers, namely, the different buoyancy due to the different densities of the composites and the neat polymer, the different thermal diffusivity of the composites and the fact that the mass loss (or remaining mass) of the composites, conventionally, is expressed per overall mass of the composite and not per mass of polymer. The relative contributions of these factors are evaluated and it is found that the conventional expression of mass loss has the most profound effect. Furthermore, it is shown that it is proper to express and evaluate the TGA results of composite polymers per degradable (polymer) mass of the composite and not per overall mass of the composite.

## 1. Introduction

Thermogravimetric analysis (TGA) is one of the most common thermos-analytical techniques and is used in a wide variety of scientific fields including foods [1], pharmaceuticals [2], the study of biofuels and energy production [3], energy storage [4], adsorption processes [5], etc. Furthermore, TGA is the primary method used for the evaluation of the thermal stability of materials [6,7]. As such, TGA is widely used for the characterization of polymeric composite materials [8,9,10].

The development of composite materials via the dispersion of inorganic and carbonaceous fillers into a polymer matrix is a very attractive approach which is used in order to enhance various properties of polymers, including the one of thermal stability. Two review articles (with the title “Can nanoparticles really enhance thermal stability of polymers?”) [11,12] nicely highlight, summarize and discuss the controversial and contradictive results and interpretations regarding the influence of nanoparticles on the thermal stability of composite polymers, which ranges from severe improvement to no influence or even to a deterioration of the thermal stability and acceleration of the thermal degradation [11,12].

In general, in the science of composite polymers, the existence of compatibility between the polymer and the filler, which leads to a good dispersion of the particles into the polymer matrix, is considered to be of primary importance for the enhancement of the properties of the polymer. For this reason, a rather large portion of the research regarding polymer composites has focused on finding ways for the appropriate modification of the filler and/or the polymer [13,14], in order to promote thermodynamically favored polymer–filler interactions [15]. The enhancement of the polymer’s properties due to the compatibility with filler may be true for other properties, e.g., mechanical; however, regarding the initiation of thermal degradation and the thermal stability, the following paradox can be detected within this concept.

It is recalled that strong intermolecular (non-covalent) interactions lead to the weaking of chemical bonds, e.g., it is widely known that the wavenumber/frequency of the stretching vibration of hydrogen bonded O–H groups is lower than the one of free groups [16]. More precisely, the frequency of the stretching vibration of a chemical bond is related to its force constant and its strength (or dissociation energy) [17,18]. The non-covalent interactions affect the force constant of the chemical bond and thus affect its stretching frequency and bond strength. In other words, strongly (non-covalently) bounded bonds are easier to break than weakly bounded ones. Of course, the overall thermal degradation of polymers is a complex process which includes multiple reactions and pathways and reactions between degradation products, e.g., a mechanism for enhancing the flame retardancy of polymers is based on the usage of additives which promote a fast initial decomposition leading to fast char formation and lower production of volatile products, which in turn retards further thermal oxidation of the polymer like it occurs in wood [19]. However, the initiation of thermal degradation is directly related to the bond strength of the chemical bonds which are present in the polymer. 

If we consider the composite material as a mixture of two components A (polymer) and B (filler), then in the case of the existence of thermodynamically favored interactions between A and B, it follows that the Gibbs free energy of mixing (Δ*G_mix_*) of A and B is negative. There are two main possibilities (excluding the combinations of them) in order to produce a negative Δ*G_mix_*: (a) the mixing of A and B leads to an increase in entropy; that is, the entropy of mixing (Δ*S_mix_*) is positive though there are no favorable A–B interactions. (b) The mixing of A and B leads to a decrease in enthalpy; that is, the enthalpy of mixing (Δ*H_mix_*) is negative; that is, A–B interactions are favored over A–A interactions. In the first case, the chemical bonds of the polymer should not be affected by the presence of the filler and thus the bonds of the polymer are expected to exhibit the same strength in the pure form and in the composite material and thus no alteration of the thermal stability (initiation of thermal degradation) would be expected to occur by the addition of the filler. In other words, if the mixing of the polymer and the filler is favored due to entropic reasons (or if there is no mixing at all and the dispersion is very poor), then no alteration of the thermal stability should be expected. In the second case, from the negative value of Δ*H_mix_* it follows that the A–B intermolecular (non-covalent) interactions are stronger than the respective A–A ones. Thus, in the composite material, the chemical bonds of the polymer are expected to be weaker than in the pure form due to the stronger intermolecular bonding. In other words, if the mixing of the polymer and the filler is favored due to enthalpic reasons, a deterioration of the thermal stability of the polymer in the composite material should be expected. Thus, it is rather a paradox to expect that the particles of the filler, in any case, could increase the bond strength of the polymer’s bonds and consequently its thermal stability. 

However, commonly, the composite materials exhibit quite different behavior to pure polymers, featuring either improved or deteriorated thermal stability. If the filler cannot alter the bond strength and the thermal stability of the polymer, then why is it common to detect differences between the behavior of the pure and the composite polymers? A possible answer could be that there are various factors in a TGA measurement that can cause apparent (fake) alterations of the thermal stability. The scope of this work is to study the effect of these factors on the thermal stability of composite polymers. Specifically, the objective of this work is to simulate the TGA signal during isothermal TGA measurements of composite polymers by assuming that the polymer exhibits the same degradation profile in the neat and the composite form (that is, it is assumed that there is no actual alteration of the thermal stability of the polymer). In such a case, no differences should be detected in the TGA curves of the neat polymer and the composite polymers. We demonstrate that this is not the case and that apparent alterations exist which have been long misinterpreted as actual alterations. To the best of our knowledge, such apparent alterations of the thermal stability of composite polymers have never been discussed and reported in the literature. 

## 2. Materials and Methods

Polyvinyl alcohol (PVA) (M_w_ = 89,000–98,000 g/mol, degree of hydrolysis > 99%) and graphite (99%, 325 mesh particle size > 99%, natural) were purchased from Sigma-Aldrich (Burlington, MA, USA). Nanotalc (average particle size < 100 nm) was obtained from Nanoshel LLC (Wilmington, DE, USA). A Shimadzu thermogravimetric analyzer (Shimadzu TGA-50, Tokyo, Japan) and a Sartorius scale (±0.0001 g, Sartorius AG, Göttingen, Germany) were used. 

PVA was dissolved in water at a concentration of 9.1 wt.% (4 g of PVA in 40 mL of water) by stirring and heating at 70–80 °C. This solution was divided into three parts of 10 g. In the first solution, no addition of filler was performed, while in the second and third solution, appropriate amounts (0.091 g) of talc and graphite were added so as to obtain 10% composition of the filler with respect to the polymer mass. The three solutions were cast in petri-dishes of identical size and were left at room temperature for 3 days in order for water to evaporate, to obtain the neat PVA film and the PVA-10% talc and PVA-10% graphite composite films. TGA isothermal measurements of these three samples were conducted at 210 °C for 15 min in a nitrogen atmosphere (flow 20 mL/min). The temperature of 210 °C was reached with a heating rate of 20 K/min. The reasons for studying composites with 10% filler are the following: (a) higher contents of filler, e.g., 30%, are not very common and are typically used in micro-composites and not nanocomposites and (b) for composites with lower filler contents, e.g., 1%, the differences may not be detectable by our TGA due to the existence of noise.

## 3. Theoretical Analysis

### 3.1. General Concepts

There are at least the three factors that can potentially cause apparent alterations of the thermal stability of composite polymers:

(1) Recently, the latent limit of detection of TGA was reported [20]. Briefly, by the subtraction of the blank TGA measurement we can take into account the error that is introduced in the measurement due to the buoyancy which is exerted on the pan. However, the buoyancy exerted on the sample always influences the TGA signal (that is, the apparent mass sample which is equal to the real mass minus buoyancy) and introduces an additional error in the detection and quantification limits of TGA [20]. Typically, the fillers that are used in polymer composites have higher densities than those of the polymers. Thus, the pure and the composite polymers have a non-negligible difference in density. Due to the different density, the buoyancy exerted on the pure and composite polymers is different, thus the limit of detection and quantification is different. In other words, due to the difference in density, the detection and quantification of mass loss appears to be different and causes an apparent difference in the thermal stability.
(2) It is widely known that in TGA, the measured temperature is not the actual temperature of the sample but the temperature of the purge gas near the sample. In addition, the sample does not have a uniform temperature but instead a temperature gradient exists within the sample. In general, such effects are well-known, e.g., that a thicker sample will exhibit a more intense temperature gradient than a thinner sample. The temperature gradient does not depend only on the dimensions of the sample but also on its thermal diffusivity (which in turn depends on the thermal conductivity, density and specific heat capacity). Thus, fillers with high and low thermal diffusivity are expected to cause, respectively, a faster and slower heating of the composite material. This is an actual and not an apparent effect, e.g., if a composite material with low thermal diffusivity is exposed in a real application at high temperature, it will be heated slower than the pure polymer. However, it is not accurate to claim that the composite material exhibits increased thermal stability. If there is no interaction between the polymer and the filler, or the compatibility arises from entopic reasons (as discussed in Section 1), the polymer will degrade at the same temperature both in the pure form and the composite material. Put simply, due to the lower thermal diffusivity of the composite material, the polymer will reach this temperature slower. In an isothermal TGA measurement, this will be observed as degradation of the composite at higher times than the pure polymer. In a non-isothermal TGA measurement, since the actual temperature of the sample is not measured, this will be observed as degradation of the composite at higher temperatures. In both cases, this may be (erroneously) interpreted as an actual increase of the thermal stability.
(3) The mass loss (or the remaining mass) of the composite materials, conventionally, is expressed per overall composite mass and not per degradable (polymer) mass of the composite. This can cause an apparent alteration of the thermal stability for the following reason: For sake of simplicity, this will be discussed through an example. If the mass sample of the pure polymer that is used for the TGA measurement is 5 mg with a scale readability of 0.01 mg, then, by excluding any other sources of error/uncertainty, the minimum % detectable mass loss is 100 × 0.01/5 = 0.2%. If the mass of the composite is also 5 mg, then the minimum detectable % mass loss is again 0.2%. However, this is true only if the mass loss arises from the overall mass sample. However, in the composite material, e.g., with 20% inorganic (non-degradable) filler, the mass loss is not due to the overall mass sample (5 mg) but only due to the degradable (polymer) mass, i.e., 5 − 5 × 0.2 = 4 mg. Thus, in the composite material, the absolute mass loss of 0.01 mg that is due to the 4 mg of degradable mass is expressed and reduced with respect to the overall mass of 5 mg. Since both measurements are conducted with the same readability, in the case of the composite material, the actual minimum detectable % mass loss is 100 × 0.01/4 = 0.25%. In other words, the TGA has the same sensitivity in detecting absolute mass loss but is less sensitive in detecting relative (%) mass loss in the composite material. This causes the composite material to appear more thermally stable than the pure polymer if the mass loss is expressed per overall mass of the composite.

It should be stressed that these three factors are always present and influence the TGA results independently of the existence of other effects, e.g., a high interaction between the polymer and the filler which may alter the chemistry of degradation of the polymer in the composite material. In order to “isolate” and study the effect of the three abovementioned factors, we performed a simulation study by assuming that there is no interaction between the filler and polymer; that is, the degradation profile of the polymer is assumed to be exactly the same in the pure form and in the composite material. In what follows, we describe the developed methodology for studying these effects.

### 3.2. Modelling

For the modelling and simulation of the TGA signal during the isothermal measurement of composite polymers, the following assumptions were made:

(1) The weight, buoyancy and drag force of the TGA pan are not taken into account since their effect can be eliminated by the subtraction of the empty pan measurement.
(2) The drag force that is exerted on the sample is considered to be low and to have a negligible effect on the TGA signal, and thus it is not taken into account in the modelling equations.
(3) There is no interaction between the polymer and the filler and thus the degradation profile and degradation temperature of the polymer are the same both in the neat and composite form; that is, the same equation describes the degradation profile of the real mass loss of the neat and composite polymer.
(4) The density, the specific heat capacity and the thermal conductivity of the polymer and the additive do not change with temperature.
(5) The density, the specific heat capacity and the thermal conductivity of the polymer do not change due to degradation. This assumption may be considered to be valid for low degrees of degradation. For this reason, the simulated results will be presented for low degrees of degradation, namely, up to 2% mass loss (98% remaining mass).

The thermal diffusivities of the polymer, the fillers and the composites were calculated from the following equation:

(1) $$\alpha =\frac{k}{d\times C_{p}}$$

where

α: thermal diffusivity,

k: thermal conductivity,

d: density,

$C_{p}$: specific heat capacity.

The thermal conductivities, densities and specific heat capacities of the composites were calculated from the following equations:

(2) $$k_{composite}=\left({1-X}_{V,filler}\right)\times k_{polymer}+X_{V,filler}\times k_{filler}$$

(3) $$d_{composite}=\left({1-X}_{V,filler}\right)\times d_{polymer}+X_{V,filler}\times d_{filler}$$

(4) $$C_{p,composite}=\left({1-X}_{W,filler}\right)\times C_{p,polymer}+X_{W,filler}\times C_{p,filler}$$

where

$X_{V,filler}$: the volume fraction of the filler in the composite material,

$X_{W,filler}$: the mass fraction of the filler in the composite material.

The volume fraction of the filler was calculated from the following equation:

$$\;X_{V,filler}=\frac{V_{filler}}{V_{filler}+V_{polymer}}\Rightarrow$$

(5) $$X_{V,filler}=\frac{\frac{m_{filler}}{d_{filler}}}{\frac{m_{filler}}{d_{filler}}+\frac{m_{initial,\;polymer}}{d_{polymer}}}$$

where

$V_{filler}$: the volume of the filler in the composite material,

$V_{polymer}$: the volume of the polymer in the composite material,

$m_{filler}$: the mass of the filler in the composite material,

$m_{initial,\;polymer}$: the initial mass of the polymer in the composite material.

The above-mentioned masses are calculated from the initial overall real mass of the sample and the mass fraction of the filler, as follows:

(6) $$m_{filler}=X_{W,filler}\times m_{overall}$$

(7) $$m_{initial,\;polymer}=m_{overall}-m_{filler}$$

where

$m_{overall}$: initial overall real mass of the composite sample.

The temperature gradient of the sample as a function of time and distance was calculated from the following equation:

(8) $$\frac{T\left(x,t\right)-T_{S}}{T_{0}-T_{S}}=\frac{4}{\pi }*\sum_{j=1,3,5\ldots }^{\infty }\frac{1}{j}*\left[\sin\left(\frac{j*x*\pi }{L}\right)*exp\left(-{\left(\frac{j*\pi }{L}\right)}^{2}*\alpha *t\right)\right]$$

where

$T\left(x,t\right)$: the temperature of the sample at any given time and distance;

$T_{S}$: the temperature of the surface of the sample (that is, at *x* = 0), which is considered to be equal to the temperature of the purge gas (nitrogen) that is used for the measurement;

$T_{0}$: initial temperature of the sample;

L: thickness of the sample;

x: distance from the sample’s surface;

α: thermal diffusivity of the sample.

It is worth mentioning that Equation (8) is valid for heat transfer in one dimension (*x*). This is true for polymer films where the one dimension (thickness) is much smaller than the other two dimensions (length and width). For the modelling we used the parameters of PVA. In order to describe the full degradation of polymers, a reaction model is required [20]. However, as discussed in the above-mentioned fifth assumption, in this work, we are interested in low degrees of conversion, e.g., up to 2%. According to a previous work, the degree of degradation of PVA (during isothermal conditions) and for mass loss up to 4–5% is linear with time and can be described without using a reaction model [21]. Thus, for the calculation of the degree of “real” degradation of the polymer, the following equation was used [21]:

(9) $$a_{real,\;polymer}(t)=K(T)\times t$$

where

$\alpha_{real,polymer}(t)$: the degree of real degradation (mass loss) of the polymer,

K(T): the reaction rate,

t: time.

The reaction rate was calculated from the Arrhenius equation; that is [21],

(10) $$K\left(T\right)=A*exp\left(-\frac{E_{a}}{R*T(x,t)}\right)$$

where

A: the pre-exponential factor,

$E_{a}$: the activation energy,

R: universal gas constant.

The real (remaining) mass of the polymer at any given time can be calculated from the following equation:

(11) $$m_{real,polymer}(t)=m_{initial,\;polymer}*\left(1-a_{real,\;polymer}(t)\right)$$

where

$m_{real,polymer}(t)$: the real (remaining) mass of the polymer at any given time.

The real mass of the composite at any given time is equal to the real mass of the polymer plus the mass of the filler; that is,

(12) $$m_{real,composite}\left(t\right)=m_{initial,\;polymer}*\left(1-a_{real,\;polymer}\left(t\right)\right)+m_{filler}$$

where

$m_{real,\;composite}(t)$: the real (remaining) mass of the composite at any given time.

The apparent mass of the composite (that is, the TGA signal) is the real mass of the composite minus buoyancy; that is [22],

(13) $$TGA\;signal=m_{apparent,composite}\left(t\right)=m_{real,composite}\left(t\right)-m_{real,composite}\left(t\right)\times \frac{d_{gas}(T)}{d_{composite}}$$

where

$m_{apparent,\;composite}(t)$: the apparent (remaining) mass of the composite at any given time,

$d_{gas}(T)$: the density of the purge gas (e.g., nitrogen) that is used for the measurement.

In Table 1, the thermal conductivities, densities, specific heat capacities and the calculated thermal diffusivities of PVA and various fillers are presented. In Table 2, the values of the various parameters that were used for the modelling are summarized. The density of gas nitrogen in the temperature range 20–200 °C was taken from the NIST Chemistry WebBook [23].

## 4. Results and Discussion

### 4.1. Simulation Results

**Figure 1:**
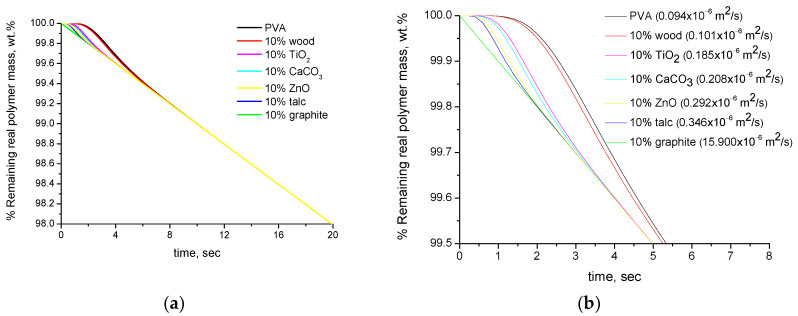
Simulated TGA curves (% remaining mass expressed per real polymer mass) of PVA composites with 10 wt.% of various fillers: (**a**) for mass loss 0–2 wt.% and (**b**) for mass loss 0–0.5 wt.% (the values of the thermal diffusivities of the composite samples are included in the legend).

In Figure 1a, the simulated TGA curves (% remaining mass expressed per real polymer mass) of PVA composites with 10 wt.% of various fillers are presented. As can be seen, after 8–10 s, all the curves coincide. This is expected since it was assumed that there is no interaction between the polymer and the fillers and that the polymer exhibits the same degradation profile in the neat and composite form. The difference of the curves up to 10 s is due to the different thermal diffusivities of the samples. In Figure 1b, the same graph as in Figure 1a is presented under different scale, and in the legend, the values of the thermal diffusivities of the composites (not the additives) have been included. As can be seen in Figure 1b, it is clear that the difference in the curves at the initial time correlates with the values of the thermal diffusivities of the samples. This is an actual effect and these samples, in reality, if exposed to 200 °C, will reach this temperature at different times, thus their curves will differ in TGA. However, this should not be interpreted as a difference in the thermal stability, since the polymer degrades with the same rate at this temperature in all cases. The time of 8–10 s is the time that is required for the sample to reach the temperature of 200 °C. After this temperature is reached, the curves of all samples, as mentioned above, coincide. It is worth mentioning that the differences in the initial stage due to the different thermal diffusivity values are countable and in the range 0.1–1 wt.%. These calculations, as mentioned in Table 2, were performed for a distance of $x=\frac{L}{4}$ for samples of thickness 1 mm. Obviously, for the center of the sample ($x=\frac{L}{2}$) or for thicker samples, the above-mentioned differences will be larger but of the same order of magnitude.

**Figure 2:**
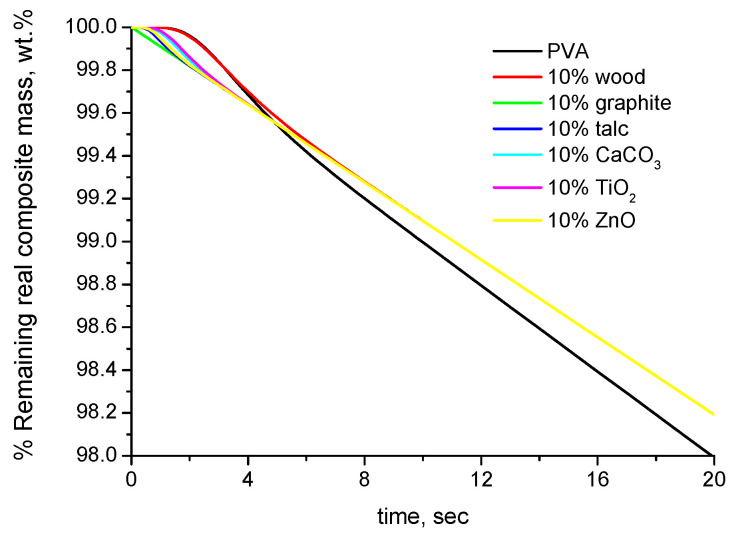
Simulated TGA curves (% remaining mass expressed per real overall composite mass) of PVA composites with 10 wt.% of various fillers.

As discussed in a previous section, the TGA results of the composites are conventionally expressed per overall composite mass and not per polymer mass, as in Figure 1. If we express the very same results of Figure 1, not per real polymer mass but per real overall composite mass, then a different behavior is observed, as shown in Figure 2. At times up to 10 s, the behavior is the same as that in Figure 1, and similar differences can be observed due to the different values of thermal diffusivity. However, after a short time, all the composite samples appear to be more stable (to degrade with a slower rate) than the pure polymer. This is a completely apparent effect due to the expression of the mass loss per overall composite mass instead of polymer mass. This effect is influenced by the mass fraction of the filler $X_{W,filler}$, and since all the samples have the same value of $X_{W,filler}$, the curves of all the composites coincide after they reach the same temperature (around 8–10 s).

**Figure 3:**
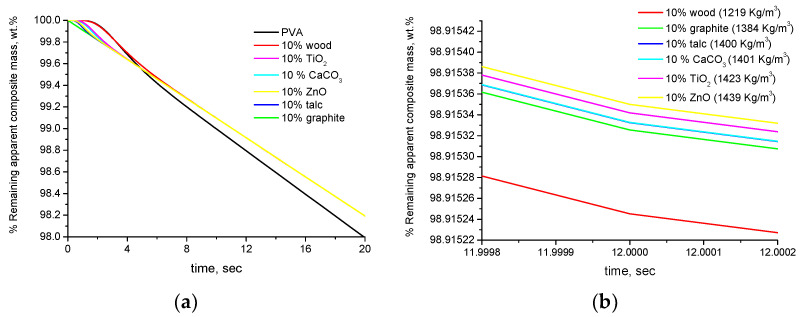
Simulated TGA curves (% remaining mass expressed per apparent overall composite mass) of PVA composites with 10 wt.% of various fillers: (**a**) for mass loss 0–2 wt.% and (**b**) mass loss at 12 s (the values of the densities of the composite samples are included in the legend).

By taking into account the different densities of the samples and thus the different buoyancies, we can express the results per apparent overall composite mass (not real mass). These TGA curves are shown in Figure 3a. The curves are practically the same as the ones of Figure 2. Only under a very different scale, a negligible difference can be observed, as shown in Figure 3b. As can be seen, these differences are of the order of magnitude of 10^−4^ wt.% and correlate with the density of the composite samples, which is presented in the legend of the figure.

Before proceeding, it is worth mentioning that the different thermal diffusivities of the various samples affects the initiation of decomposition. For example, the graphite sample is heated faster and thus initiates mass loss at t = 0 s. The same effect may arise from different heating rates. For example, at low heating rates, the samples with high thermal diffusivity may start to decompose before the constant temperature of the isothermal experiment is reached.

**Figure 4:**
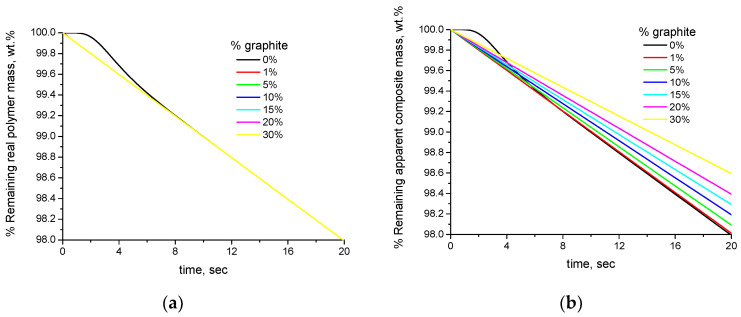
Simulated TGA curves of PVA–graphite composites of various values of $X_{W,filler}$: (**a**) % remaining mass expressed per real polymer mass and (**b**) % remaining mass expressed per apparent overall composite mass.

As mentioned above, the different slope of the apparent (or real) overall composite mass of the pure polymer and the composite samples is influenced by the mass fraction of the filler $X_{W,filler}$. This becomes obvious from the TGA curves of PVA–graphite composites of various values of $X_{W,filler}$, which are presented in Figure 4.

**Figure 5:**
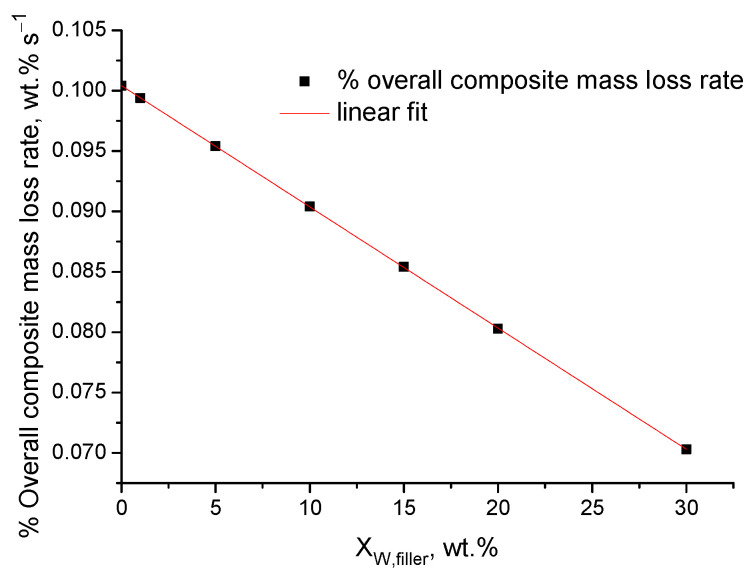
Simulated overall composite mass loss rate of PVA–graphite composites versus $X_{W,filler}$ and linear fit.

Figure 4a,b present, respectively, the simulated TGA curves of the PVA–graphite composites expressed per real polymer mass and per apparent overall composite mass. As can be seen, despite the fact that the mass loss of the real polymer mass is the same (Figure 4a), if expressed per overall composite mass, the samples appear to be countably more stable than the pure polymer when increasing the value of $X_{W,filler}$. It is worth mentioning that this apparent increase of the thermal stability of the composites, i.e., the lower mass loss rate of the apparent overall composite mass (after 10 s) of the composite samples, is directly proportional to the value of $X_{W,filler}$, as shown in Figure 5.

As a summary of this section, the different densities and thermal diffusivities of the composites and the conventional expression of the TGA results per overall composite mass cause apparent alterations of the thermal stability of composite polymers even in the case in which the degradation actually occurs at the same temperature with the same rate. The effect of buoyancy due to the different values of the densities of the composites samples is negligible and in the order of magnitude of 10^−4^ wt.%. The effect of slower or faster heating is an actual effect; however, it should not be interpreted as a difference in the thermal stability. The effect of the thermal diffusivity is orders of magnitude higher than the one of density and of the order of magnitude of 10^−1^ wt.%; however, it vanishes fast and its severity can be reduced by using samples of small thickness. The conventional expression of the results per overall composite mass has the most profound effect, since it is of the order of magnitude of 10^−1^ wt.%, and not only does it not vanish but it grows with time, since it affects the apparent overall composite mass loss rate which is directly proportional to the value of $X_{W,filler}$.

Finally, it is worth mentioning that similar apparent alterations of the thermal stability of composite polymers are expected to exist also in non-isothermal TGA measurements. However, the simulation of non-isothermal TGA measurements is more complicated, especially the part regarding the calculation of the temperature gradient of the sample. Specifically, Equation (8) refers to the case of the temperature gradient of the sample if exposed to a constant temperature (isothermal TGA) and not a temperature ramp (non-isothermal TGA). Thus, the solution of the Fourier equation for such cases of heat transfer and the simulation of the non-isothermal TGA measurement requires different handling and deserves a future dedicated study.

### 4.2. Experimental Results

It should be stressed that a direct comparison of the experimental and simulated results is of low value for the following reasons. First, the modelling equation for the heat transfer (Equation (8)) refers to a different problem to the actual TGA measurement. Specifically, in the modelling it was assumed that the samples are exposed directly at 200 °C while in the actual TGA measurements they are exposed to a temperature ramp. Second, the thermal behavior of PVA in the form of raw powder and film has recently been thoroughly studied [21,27]. Briefly, PVA exhibits two mass loss stages, one at lower temperatures up to 130 °C and one that initiates at around 210 °C. In the film form, the end of the first stage of mass loss interferes with the beginning of the second stage of mass loss.

**Figure 6:**
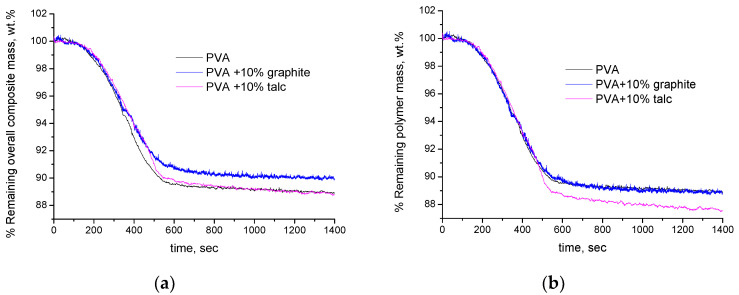
Experimental TGA curves of PVA, PVA + 10% graphite, and PVA + 10% talc composites: (**a**) % remaining mass expressed per overall composite mass and (**b**) % remaining mass expressed per polymer mass.

In Figure 6a,b, the experimental curves of neat PVA, PVA + 10% graphite and PVA + 10% talc composite films are presented, respectively, per overall composite mass and per polymer mass. As can be seen in Figure 6a (per overall composite mass), and based on the established interpretations, we would say that the composites exhibit increased thermal stability compared to the neat polymer. Furthermore, the composite PVA–graphite sample seems to be more stable than the one with talc. We are unable to provide an explanation for such conclusions. However, if we express the mass loss per polymer mass (Figure 6b), a different and rather easily explainable behavior is observed. As can be seen, the curves of the neat PVA and the PVA + 10% graphite composite films practically coincide. This agrees with the simulation analysis which was presented in the previous sections and points out that the degradation profiles of PVA in the neat and composite form are the same. This is reasonable, since no serious interaction between PVA (highly polar groups) and graphite (low polarity) is expected; thus, no alteration of the bond strengths (and thus thermal stability) of PVA is expected in the neat and composite form. On the contrary, as can be seen in Figure 6b, the PVA + 10% talc composite film, regarding the second stage of mass loss which initiates around 800 s, is less thermally stable and degrades with a higher rate than pure PVA. It is worth mentioning at this point that since we study low degrees of mass loss and the differences among the samples are low, we prefer not to smooth the data but to use the raw data in order to avoid any alteration of the slopes, etc. Specifically, the mass loss rate after 800 s of pure PVA and PVA + 10% graphite is 0.037 wt.%/min while the respective one of the PVA + 10% talc is 0.057 wt.%/min. This is also reasonable since strong ion–dipole interactions are expected to occur between PVA and talc. The stronger ion–dipole interactions in the composite sample (compared to the dipole–dipole interaction in the neat PVA) lead to the weakening of chemical bonds of PVA. It is known that the activation energy is related to the bond strength of the reactants. Thus, the weaker chemical bonds are translated to lower activation energy and thus faster reaction rate.

**Figure 7:**
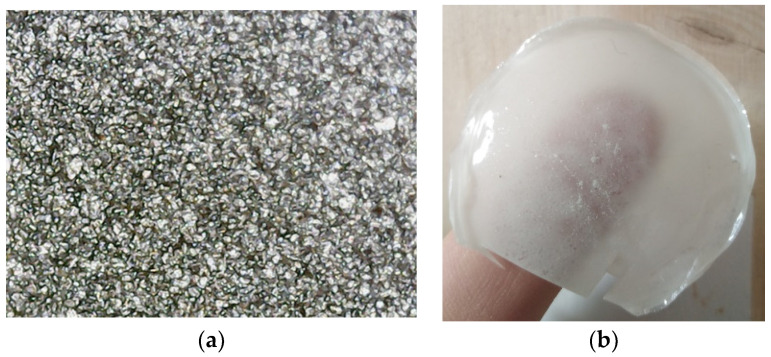
Images of the composite samples: (**a**) image from digital optical microscope of the PVA + 10% graphite composite, showing the extensive existence of large aggregates; (**b**) macroscopic photo of the PVA + 10% talc composite, showing the semi-transparency of this sample.

The absence of interaction between PVA and graphite, which is theoretically justified, is also confirmed by the extensive existence of large graphite aggregates, as shown in Figure 7a. Although aggregates were visible also in the PVA + 10% talc sample, the presence of strong interactions between PVA and talc, which is theoretically justified, is also confirmed by the semi-transparency of this sample, as shown in Figure 7b.

Furthermore, it is worth mentioning that in a previous work [28] for the development of poly (propylene)-composite-drawn fibers which also contained antioxidant, we studied via Fourier transform infrared spectroscopy (FTIR) the interactions of a phenolic-type antioxidant with talc and found that the O–H stretching vibration of the antioxidant was shifted when it was blended with talc. This indicated an interaction of the O–H with talc since the observed shifting could not be solely attributed to interaction of the antioxidant with the water adsorbed on talc. Thus, the O–H groups of PVA are expected to exhibit similar interactions with talc.

Of course, although it was not taken into account in the simulation, the particle size may cause apparent alterations for the following reason: Smaller particles translate to higher surface areas, which facilitates heat transfer. Thus, for two composites of the same filler and same filler composition, the one with the lower particle size will be heated faster, as occurs with the samples with high thermal diffusivity. A dedicated work is required in order to quantify this effect.

It should be again stressed that it is a paradox to expect that a strong interaction between the polymer and the filler can result in an increase of the thermal stability of the polymer. As discussed in Section 1, if the interaction between the filler and the polymer is stronger than the polymer–polymer interaction (as expected for the case of PVA with talc since the ion–dipole interactions are typically stronger than dipole–dipole interactions), then the chemical bonds of the polymer are weakened in the composite form due to the higher stretching of the chemical bond, which is caused by the stronger intermolecular interaction in the composite form. Thus, the weaker bonds in the composite form should break easier and thus the thermal stability should be deteriorated and not be improved.

As discussed in the Introduction, the effect of particles on the thermal stability of composite polymers is not clear and often contradictive results and interpretations have been reported. The recognition of the above-mentioned paradox, as well as the other insights which were reported in this study, e.g., the expression of the TGA results per polymer mass and not per overall mass, could assist in solving some of these contradictions.

## 5. Conclusions

The observed differences of the neat and composite polymers in TGA measurements are to some extent apparent. There are at least three factors which can cause apparent alterations of the thermal stability of composite polymers, even in the case in which the degradation of the polymer actually occurs at the same temperature with the same rate. Namely, these factors are the different density of the pure polymer and the composite samples, the different thermal diffusivity of the pure polymer and the composites, and the conventional expression of the TGA results per overall composite mass and not per degradable (polymer) mass. The different density has as a consequence the exertion of different buoyancies on the samples and causes changes in the apparent mass of the samples as measured by TGA. However, this effect causes very small differences of the order of magnitude of 10^−4^ wt.%. The effect of the different thermal diffusivity of the samples is much higher and of the order of magnitude of 10^−1^ wt.%; however, it vanishes after a short time and when the actual temperature of the samples becomes equal to the temperature measured by the TGA (the temperature of the purge gas) during an isothermal TGA measurement. Of the same order of magnitude (10^−1^ wt.%) is the effect of the conventional expression of the TGA results per overall composite mass. However, this third factor has the most profound effect, and it does not vanish with time like the effect of thermal diffusivity. On the contrary, it grows with time and is proportional to the mass fraction of the filler. For this reason, it is proper to express the TGA results of composite polymers per mass of polymer and not per overall composite mass, as confirmed experimentally.

## Figures and Tables

**Table 1:** Density, specific heat capacity, thermal conductivities and calculated values of thermal diffusivity for the studied materials. All data were taken from reference [24] unless otherwise noted.

Material	d, kg/m^3^	Cp, J/g·K	k, W/m·K	a, m^2^/s
PVA	1329 [25]	1.6 [25]	0.2 [25]	9.41 × 10^−8^
Wood	700 [26]	1.45 [26]	0.17 [26]	1.68 × 10^−7^
Graphite	2200	1.17	541.61	2.10 × 10^−4^
Talc	2700	0.85	10.6	4.63 × 10^−6^
CaCO_3_ (Calcite)	2720	1.02	5.05	1.82 × 10^−6^
TiO_2_	3900	0.68	5.6	2.11 × 10^−6^
ZnO	5600	0.62	17	4.90 × 10^−6^

**Table 2:** Values of the various modelling parameters. The values were set arbitrarily except the ones based on literature data, as noted.

Parameter	Value
Polymer	PVA
Filler	wood, graphite, talc, CaCO_3_, TiO_2_ and ZnO
$m_{overall}$	5 mg
$X_{W,filler}$	0, 1, 5, 10, 20 and 30 wt.%
$T_{S}$	200 °C
$T_{0}$	20 °C
L	1 mm
x	$\frac{L}{4}$
A	2.599 × 10^9^ s^−1^ [21]
$E_{a}$	110 KJ/mol [21]

## Data Availability

Data available upon request (due to privacy).
